# A Prospective Comparative Study Evaluating Functional Outcomes in Adolescents Undergoing Dynamic Compression Plating Versus Intramedullary Nailing for Both-Bone Diaphyseal Forearm Fractures

**DOI:** 10.7759/cureus.87451

**Published:** 2025-07-07

**Authors:** Praveenkumar Udayakumar, Bhuvanesh Gopal, Dharmveer kumar Dubey

**Affiliations:** 1 Department of Orthopedic Surgery, Jawaharlal Institute of Postgraduate Medical Education and Research, Puducherry, IND; 2 Department of Orthopaedics, Indira Gandhi Government General Hospital and Post Graduate Institute, Puducherry, IND

**Keywords:** adolescent forearm fracture, both bone forearm fractures, dynamic compression plating, grace and eversmann score, intramedullary nailing, tens nailing

## Abstract

Aim

To compare the functional outcomes of dynamic compression plating and intramedullary nailing in adolescents (12-19 years) with both-bone diaphyseal forearm fractures using the Grace and Eversmann scoring system.

Materials and Methods

Fifty-six adolescents (aged 12-19 years) with both-bone diaphyseal forearm fractures and meeting the inclusion criteria were enrolled in this study. Patients were assigned consecutive numbers; those with even numbers underwent intramedullary nailing, while those with odd numbers received dynamic compression plating. Functional outcomes were assessed at six months postoperatively using the Grace and Eversmann scoring system.

Results

Out of the 56 patients, the mean Grace and Eversmann score for the dynamic compression plating and intramedullary nailing groups was 9.79 and 9.75, respectively, at the final postoperative follow-up which was done after six months. An interpretation of the scores at the sixth month follow-up also revealed that in the dynamic compression plating group, 78.5% of patients achieved 'EXCELLENT' results and 21.5% achieved 'GOOD' results, whereas in the intramedullary nailing group, corresponding results were seen in 75% and 25% of the participants, respectively.

Conclusion

Both modalities were equally effective in the fixation of both-bone forearm fractures in adolescents aged 12-19 years, with no statistically significant difference in the functional outcome between the two groups.

## Introduction

Pediatric both-bone diaphyseal forearm fractures constitute 14.9% of all pediatric bone fractures, with the most common etiology being a fall on an outstretched hand [1]. Both bones of the forearm as a structural unit play an important role in key movements involving flexion and extension at the elbow joint, as well as supination and pronation at the radioulnar joints. Hence, restoration of normal anatomy and biomechanics is essential in these fractures to prevent deformities. A 100 degrees of elbow flexion and forearm rotation are required to accomplish the activities of daily living [2].

Fractures of both bones of the forearm are considered intra-articular injuries since the forearm is a quadrilateral joint, including the proximal radioulnar joint (PRUJ) and distal radioulnar joint (DRUJ). Hence, any disruption affects the quadrilateral ring [3]. Diaphyseal radius fractures are defined as fractures occurring between the neck of the radius proximally and the metaphyseal-diaphyseal junction distally, which is approximately 3 cm proximal to the distal articular surface. Diaphyseal ulna fractures are defined as those occurring between the distal aspect of the coronoid proximally and the ulnar neck distally [4].

Treatment of diaphyseal both-bone forearm fractures involves the following points: obtaining adequate fracture reduction, preserving biology, maintaining fracture reduction, and allowing an early range of motion. Acceptable limits of angulation for diaphyseal both-bone forearm fractures are 20° angulation (distal third), 15° (middle third), and 10° angulation (proximal third). If the shortening is less than 1 cm, even up to 100% of the translation is acceptable [5]. Accepted surgical treatment modalities for the fixation of the forearm shaft fractures are broadly divided into intramedullary nailing, open reduction, and plate osteosynthesis [6]. It is well known that children older than 10 years have less remodeling potential than children less than 10 years of age [7]. In the case of older children with less than two years of remaining growth, adult criteria are employed because of their reduced fracture remodeling potential [5].

Intramedullary nailing is a minimally invasive technique for the fixation of these forearm shaft fractures but requires limb immobilization until early callus formation is seen radiologically, despite the theoretical advantage of rotational stability with modern interlocking nails. For the placement of a nail of 3 mm diameter or more, aggressive reaming will be required, potentially resulting in thermal necrosis, jeopardizing bone viability, additional comminution, and incarceration of the nail [8,9]. In intramedullary nailing, fracture site gapping can occur during canal preparation and passage of the prebent nails. Hence, this step must be done carefully.

Open reduction and plate osteosynthesis is a widely used method for treating unstable forearm fractures [10,11]. Removal of the hardware has several risks, with reported re-fracture rates as high as 18% [12], with re-fracture occurring either through one of the empty screw holes or through the original fracture site. Although plate fixation allows more anatomic and stable correction with restoration of the radial bow [13], unsightly scars and muscle fibrosis with subsequent motion loss are noted when compared with minimally-invasive procedures like intramedullary nailing [14].

Surgical management of both-bone forearm fractures in adolescents presents a unique challenge due to the considerable variability in bone remodeling capacity within this age group. The current literature lacks consensus regarding the optimal treatment approach: whether to employ plate fixation, which is commonly recommended in adults, or intramedullary nailing, a technique generally favored in younger children. Adolescents represent a transitional age group in which both methods are frequently utilized, each associated with distinct advantages and limitations. Accordingly, a prospective comparative study was undertaken to evaluate and compare the functional outcomes of dynamic compression plating and intramedullary nailing in the treatment of adolescent both-bone forearm fractures.

Study objectives

Our primary objective was to study, evaluate, and compare the functional outcome and complications between the two methods of internal fixation (dynamic compression plating vs. intramedullary nailing) in both-bone diaphyseal fractures of the forearm using the Grace and Eversmann scoring system in adolescents between 12 to 19 years of age. Our secondary objectives were to study the modes of injury resulting in such fractures, the fracture site, and the pattern of these fractures, as well as the tourniquet and surgery time for the procedures.

## Materials and methods

Study design

The study was designed as a prospective comparative study and conducted between September 2019 and May 2021. The sample size was calculated using G*Power software (Version 3, Heinrich-Heine-Universität Düsseldorf, Düsseldorf, Germany) [15] and estimated as 28 patients in each group with an effect size of 0.8 (large effect) at a 5% level of significance and 90% power. Before clinical commencement, the study was registered with the Institutional Ethics Committee of the Indira Gandhi Government General Hospital & Post Graduate Institute (IGGGH & PG Institute) (approval no: GHSAC/2019).

Setting and participants

Participants included in the study were patients admitted with diaphyseal both-bone forearm fractures in the Department of Orthopaedics, IGGGH & PG Institute, Puducherry, from September 2019 to May 2021. These patients underwent either dynamic compression plating or intramedullary nailing after meeting the selection criteria.

Inclusion criteria

The study included male and female patients aged between 12 and 19 years. Patients with closed/open type 1 both-bone diaphyseal forearm fractures, and patients with a history of recent injury (less than one month).

Exclusion criteria

The study excluded patients with associated head injuries, those with Gustilo-Anderson [5] open type II and III fractures, and patients with local tissue conditions that make surgery inadvisable, such as infected wounds or other neuromuscular disorders. Other exclusion criteria included patients with Galeazzi or Monteggia fractures, dislocation or single forearm bone fractures, those with fractures of other bones in the ipsilateral limb, patients with any distal neurovascular deficit, and those with mental or physical inability to cooperate.

Preoperative management

Patients satisfying the selection criteria were numbered continuously and divided into two groups: odd-numbered patients were treated with dynamic compression plating (Group A), while even-numbered patients were treated with intramedullary nailing (Group B). Informed written consent was obtained from either a parent or local guardian for all study participants satisfying the inclusion criteria. They were informed about the study conditions, objectives, advantages, and risks associated with the study. The diagnosis was made after a thorough clinical and radiographic assessment, and the Arbeitsgemeinschaft für Osteosynthesefragen/Orthopaedic Trauma Association (AO-OTA) classification [16] was adopted. Upon admission, a long arm slab was applied after longitudinal traction, and the limb was kept elevated in the ward to prevent swelling. Preoperative investigations were conducted, and pre-anesthetic evaluation was performed before surgery. All surgeries were done under regional or general anesthesia. Penicillin allergy was ruled out with an intradermal drug sensitivity test for all patients before surgery, as per the institutional policy, as the allergic history was unknown in the case of many patients.

Surgical procedure

The patient was positioned supine on the operating table with the affected arm placed on a side table. Preoperative antibiotics were given 20 minutes before the skin incision. A pneumatic tourniquet was applied to the arm with the pressure set at 50-100 mm Hg above the systolic blood pressure, typically not exceeding two hours, with an absolute maximum of three hours. Pre-wash of the limb was done using a 7.5% povidone-iodine scrub solution, diluted by 50% with sterile saline. In the case of iodine allergy, hexachlorophene skin cleansers were used. The limb was then painted with 10% Betadine solution from mid-arm to fingers, draped, and cleansed with spirit.

Plate Osteosynthesis

The radius was exposed through Henry's volar approach, and the ulna was exposed through the direct approach over the subcutaneous border of the ulna. Periosteal stripping was kept at a minimum. The fractured ends of the radius and ulna were curetted by removing the clots at the ends. The fracture was reduced and fixed with a plate applied to the tensile surface of the bone. An AO dynamic compression plate (2.7 mm or 3.5 mm screw system; Hardik International Pvt. Ltd., Rajkot, India) was used depending on the bone morphology. A 2.7 mm drill bit was used for drilling the hole for a 3.5 mm cortical screw in the radius/ulna and tapped. Using a depth gauge, the length of the screw was determined and applied to fix the plate to the bone. For 2.7 mm cortical screw fixation, a 2.0 mm drill bit was used for drilling the hole. Axial compression was achieved through the oval holes, which allowed eccentric placement of the screws when required. Plates were accurately centered over the fracture site with at least six cortical purchases by screws on either side of the fracture. Wound closure was performed with 2-0 Vicryl (Lotus Surgicals Pvt. Ltd., Mumbai, India) for subcutaneous closure and 3-0 Ethilon (Ethicon, New Jersey, US)/staplers for skin closure. A sterile compressive dressing was applied, and the limb was kept elevated.

Elastic Stable Intramedullary Nailing

Titanium elastic nails (Hardik International Pvt. Ltd., Rajkot, India), which are alloys of Ti-6Al-7Nb, vary in width, according to the appropriate color coding. The ends are beak-like in shape for easy insertion. Closed reduction was attempted by traction, counter-traction, and gentle manipulation under anesthesia, guided by a C-arm. The nail size was determined under image intensifier guidance, occupying at least 2/3rd of the diameter of bone at the isthmus level. The entry point was made with an awl or a drill perpendicular to the bone initially. Once the near cortex was perforated and the medullary canal was reached, the awl was angulated around 45° to the shaft axis and advanced in oscillatory movements to create an oblique canal. Physeal-sparing entry portals were employed (at least 2 cm away from the physis). After making the entry point, the nail was inserted with the tip perpendicular to the shaft until the far cortex was felt. The nail was then rotated 180° and advanced using the curved side of the tip as a gliding aid in an oscillating maneuver. The working length between the entry point and the nail inserter was kept around 3-5 cm to enhance control during insertion. Pre-contouring of the nail was done at the fracture site. The nail was inserted with two separate nail inserters.

The appropriately sized nail was passed until it reached the fracture site. If there was difficulty with the passage, the first nail was rotated back to its initial position to facilitate the insertion of the second nail. Closed reduction was achieved by placing the tip of one nail toward the opposite fracture plane and using traction, angulation, and translation, with the nail advanced into the proximal fragment. If closed reduction was not achieved after three attempts, open reduction was employed. The posterior interosseous nerve and superficial radial nerve are at risk, especially in proximal third radius fractures. The radius fracture site was reduced through a small skin incision, and the nail was passed under direct vision after reduction. Once the radius was reduced, the ulnar fracture reduction was attempted by closed reduction. If difficulty persisted, open reduction was done, and the nail was advanced into the distal fragment. The nail tips were positioned facing each other to tension the interosseous membrane, then cut at the end to avoid their protrusion. The stability of fixation and range of motion were then assessed gently. The wound was then closed in layers, and a sterile compression dressing was applied, with limb elevation maintained. Immobilization in the form of a plaster cast or brace was given for at least six weeks in this group.

Postoperative care

Antibiotics (inj. cefotaxime 50 mg/kg IV and inj. gentamycin 2.5 mg/kg, maximum 80 mg) were given for three days. In the case of penicillin-allergic cases, clindamycin 10 mg/kg (maximum 900 mg IV) was substituted. Regular wound inspection was performed. Limb elevation was maintained using an IV stand to prevent edema and analgesics (inj. diclofenac) were given once the regional anesthesia effect wore off. Sutures were removed on the 14th postoperative day. Mobilization of the wrist, elbow, and forearm was initiated once the patient tolerated pain in the dynamic compression plating group. Immobilization in the form of a plaster, cast, or brace was provided in all intramedullary nailing cases for at least six weeks postoperatively. Active finger movements and shoulder mobilization were encouraged.

Follow-up

Patients were followed up at regular intervals of three weeks, six weeks, three months, and six months postoperatively. A clinical assessment of the range of motion in the elbow and radioulnar joints (in degrees) was performed. Further anteroposterior and lateral radiographs were taken during follow-up visits to assess callus formation at the end of six weeks and six months postoperatively to look for the maintenance of fracture reduction and implant position. All ranges of movement were measured only by the principal investigator with a goniometer to avoid bias. Functional outcomes were then graded at the final follow-up using the Grace and Eversmann scoring system (Table 1) [17].

**Table 1:** Grace and Eversmann scoring system Reproduced with permission from Wolters Kluwer Health, Inc.

Parameter	EXCELLENT (Score-4)	GOOD (Score-3)	FAIR (Score- 2)	POOR (Score-1)
Supination and pronation range of movements	>80°	60° to 80°	40° to 60°	<40°
Range of movement - elbow	120°	120° - 100°	100° - 80°	80°
Radiological union at the sixth week	Union achieved (Score-2)	-	No union achieved (Score-1)	-
Total score obtained	≥10	8 – 9	6 – 7	≤5

Statistical analysis

The data collected were entered in a Microsoft Excel (Microsoft Corp., Redmond, WA, US) document. Compiled data and variables were coded, recorded, and analyzed as per the objectives of the study. IBM SPSS Statistics for Windows, Version 23 (Released 2015; IBM Corp., Armonk, New York, United States) was used for statistical testing [18]. Normally distributed continuous variables were expressed as mean ± SD. Categorical variables were expressed as frequencies and percentages. Comparison of the radiological union, final score, range of movements (supination, pronation, elbow flexion and extension), tourniquet time, and surgery time between the dynamic compression plating and intramedullary nailing groups was done by the Student's t-test. For all statistical tests, a p-value less than 0.05 indicated a significant difference. The results were summarized and represented graphically in the form of bar diagrams.

## Results

The study involved 56 participants from the Department of Orthopedic Surgery at IGGGH & PG Institute, Puducherry, India, from September 2019 to May 2021. The study aimed to compare the functional outcomes between dynamic compression plating and intramedullary nailing in the operative management of both-bone forearm fractures in adolescents (aged 12-19 years).

**Figure 1:**
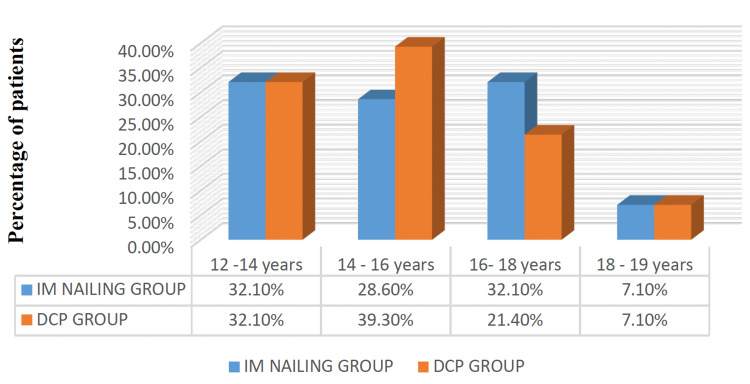
Age distribution (in years) among the 56 study participants included in both groups IM nailing: Intramedullary nailing (n=28); DCP: Dynamic Compression Plating (n=28)

Both study groups (Groups A and B), each comprising 28 patients, had a similar mean age (15.43±1.7 years and 15.57±2.15 years for dynamic compression plating and intramedullary nailing, respectively), with road traffic accidents (RTA) being the predominant cause of injury in 60.7% of the former group and 53.6% of the latter group (Figure 1).

**Figure 2:**
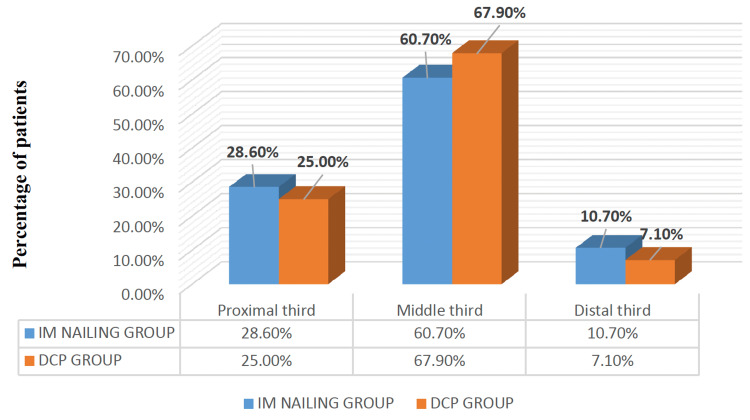
Distribution of the fracture site (%) among the 56 study participants in both groups IM nailing: Intramedullary nailing (n=28); DCP: Dynamic Compression Plating (n=28)

The middle-third shaft fractures were the most frequent fracture site in both groups (67.9% and 60.7% for the dynamic compression plating and intramedullary nailing groups, respectively), as depicted in Figure 2.

**Figure 3:**
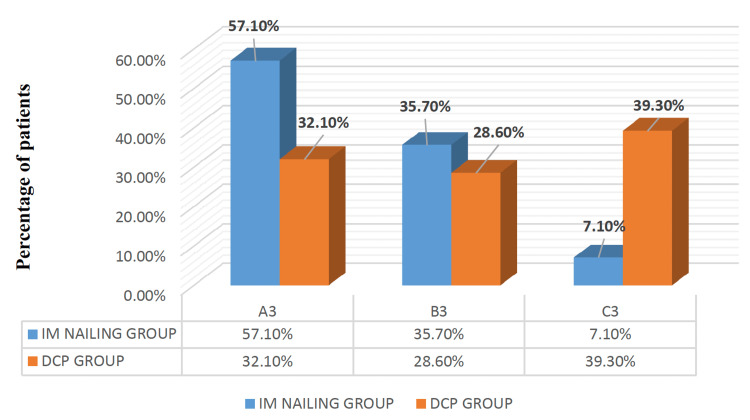
AO-OTA classification of fracture pattern among the 56 study participants in both groups IM nailing: Intramedullary nailing (n=28); DCP: Dynamic Compression Plating (n=28); AO-OTA: Arbeitsgemeinschaft für Osteosynthesefragen/Orthopaedic Trauma Association classification.

Fracture classification types revealed that closed fractures were most common in both groups, according to Gustilo-Anderson classification (78.6% and 82.1% of patients in the dynamic compression plating and intramedullary nailing groups, respectively), with Type 1 open fractures being less frequent (17.9% in the former and 21.4% in the latter group, respectively). Based on the AO-OTA classification, 22-A3 was the most common type in the intramedullary nailing group, while the 22-C3 type was predominant in the dynamic compression plating group (Figure 3).

In the sixth month of follow-up, the range of supination, elbow flexion, and extension movements achieved were comparable between both groups (p>0.05), but the range of pronation was better in the intramedullary nailing group (mean: 80.7 degrees; n=28) and statistically significant compared to the dynamic compression plating group (mean: 78.57 degrees; n=28) (p<0.05), as represented in Table 2.

**Table 2:** Degrees of radio-ulnar and elbow joint movements achieved among the 56 study participants in both groups IM nailing: Intramedullary nailing (n=28); DCP: Dynamic Compression Plating (n=28); df: degrees of freedom. Student's t-test was applied for comparison of means; p value <0.05 was considered statistically significant. *Student's t-test could not be applied for the range of elbow extension movements data as the standard deviation (SD) was zero in both groups.

Movements	IM nailing group (mean ± SD)	DCP group (mean ± SD)	Difference in mean (95% CI)	t value	df	p value
Supination (in degrees)	85.71 ± 7.16	85.71 ± 7.418	0.0 (-3.9 to 3.9)	0	54	1.00
Pronation (in degrees)	80.71 ± 3.78	78.57 ± 2.99	2.14 (0.31 - 3.9)	2.34	51	0.02
Elbow flexion (in degrees)	133.04 ± 6.8	133.3 ± 5.9	0.35 (-3.7 to 3.0)	0.15	53	0.83
Elbow extension (in degrees)	5.0 ± 0	5.0 ± 0	0.00	-	-	*Not applicable

While the intramedullary nailing group achieved radiological union slightly faster at an average of 8.5 weeks compared to 9.4 weeks for the dynamic compression plating group, this difference was not statistically significant (p>0.05). Similarly, the Grace and Eversmann scores, which assess functional outcomes were comparable between both groups with an average score of 9.7 and there was no statistically significant difference (p>0.05) noted. The intramedullary nailing group performed better, exhibiting statistically significant shorter tourniquet utilization times (mean: 41.2 minutes vs. 67.5 minutes in the dynamic compression plating group; p<0.05). Similarly, surgical procedural times were quicker for the former than the latter group (mean: 58 minutes vs. 79 minutes), respectively. This difference was also statistically significant (p<0.05), as shown in Table 3. 

**Table 3:** Difference in various outcomes among the 56 study participants included in both groups IM nailing: Intramedullary nailing (n=28); DCP: Dynamic Compression Plating (n=28); df: degrees of freedom. Student's t-test was applied for comparison of means; p value <0.05 was considered statistically significant.

Outcome	IM nailing group (mean ± SD)	DCP group (mean ± SD)	Difference in mean (95% CI)	t value	df	p value
Radiological union (in weeks)	8.5 ± 2.9	9.4 ± 1.9	0.92 (-2.2 to 0.4)	-1.37	47	0.17
Grace and Eversmann score at the sixth month of follow-up	9.7 ± 0.4	9.7 ± 0.4	0.36 (-2.6 to 0.1)	0	54	0.75
Tourniquet time (in minutes)	41.2 ± 3.9	67.5 ± 6.5	26.2 (-29.1 to -23.3)	-18.35	44	0.00
Surgical procedure time (in minutes)	58.0 ± 4.5	79.1 ± 6.2	21.0 (-24.0 to -18.1)	-14.55	49	0.00

The most frequently observed complication in both the dynamic compression plating and intramedullary nailing groups was stiffness in pronation-supination movements, present in 17.9% and 14.3% of participants, respectively. Infections (predominantly superficial) were the second most common complication encountered in 10.7% of the dynamic compression plating and 3.6% of the intramedullary nailing group. Additionally, delayed union was noted in 3.6% of the former and 10.7% of the latter group. Overall, the net complication rate was 32.1% and 28.6% in the dynamic compression plating and intramedullary nailing group (Table 4).

**Table 4:** Complications encountered among the 56 study participants included in both groups IM nailing: Intramedullary nailing (n=28); DCP: Dynamic Compression Plating (n=28). Data represented as a percentage (%) of the involved cases in each group respectively.

Complications	IM nailing group (n, %)	DCP group (n, %)
Stiffness	4 (14.3%)	5 (17.9%)
Delayed union	3 (10.7%)	1 (3.6%)
Infection	1 (3.6%)	3 (10.7%)
None	20 (71.4%)	19 (67.9%)
Total	28 (100%)	28 (100%)

Table 5 displays the summary of the observed results and the study's key findings. 

**Table 5:** Summary of the observed results among the 56 participants included in the study IM nailing: Intramedullary nailing (n=28); DCP: Dynamic Compression Plating (n=28); RTA: Road Traffic Accident; AO-OTA: Arbeitsgemeinschaft für Osteosynthesefragen/Orthopaedic Trauma Association classification.

Characteristic/Outcome	DCP group	IM nailing group
Age in years (mean ± SD)	15.43 ± 1.7	15.57 ± 2.15
RTA as a mode of injury	60.7%	53.6%
Fall as a mode of injury	32.1%	42.9%
Assault as a mode of injury	3.6%	7.1%
Fracture site: Proximal 1/3rd	25%	28.6%
Fracture site: Middle 1/3rd	67.9%	60.7%
Fracture site: Distal 1/3rd	7.1%	10.7%
Gustilo-Anderson: Closed injury	78.6%	82.1%
Gustilo-Anderson: Open type I injury	17.9%	21.4%
Most incident subtype as per AO-OTA classification	22-C3 type	22-A3 type
Mean supination (in degrees)	85.71	85.71
Mean pronation (in degrees)	78.57	80.71
Mean elbow flexion (in degrees)	133.3	133.04
Mean elbow extension (in degrees)	5.0	5.0
The mean time for radiological union (in weeks)	9.4	8.5
Grace and Eversmann score at sixth month follow up (mean score)	9.7	9.7
Mean tourniquet time (in minutes)	67.5	41.2
Mean surgical procedure time (in minutes)	79	58

These indicate that the intramedullary nailing group significantly outperformed the dynamic compression plating group in select areas. Specifically, the former group had shorter tourniquet utilization times (p<0.05), quicker surgical procedural times (p<0.05), and a better range of pronation movements (p<0.05) than the latter. Conversely, no statistically significant differences were observed between the groups regarding radiological union time (p>0.05) or the Grace and Eversmann functional scores (p>0.05).

## Discussion

The management of both-bone diaphyseal forearm fractures depends on the patient's age, expected degree of remodeling and remaining growth, fracture pattern, comminution, and any associated neurovascular injuries. The primary treatment goals are to achieve optimal fracture reduction and maintenance, promote union, and allow early mobilization to prevent deformity and loss of range of motion. In cases of irreducible, displaced forearm fractures where the displacement exceeds acceptable limits, internal fixation becomes the treatment of choice. 

Functional outcome and complications

Our study included 56 patients with both-bone diaphyseal forearm fractures who met the inclusion and exclusion criteria, consecutively numbered and treated with either plate osteosynthesis (Group A; odd-numbered) or intramedullary nailing (Group B; even-numbered). Patients were followed up at three weeks, six weeks, three months, and six months, with functional outcomes assessed using the Grace and Eversmann scoring system [17]. Callus formation and fracture union were monitored through X-rays. At final follow-up, Group A achieved 'EXCELLENT' outcomes in 78.5% and 'GOOD' outcomes in 21.5%, while Group B had 'EXCELLENT' outcomes in 75% and 'GOOD' outcomes in 25%, with mean Grace & Eversmann scores of 9.79 in Group A and 9.75 in Group B, showing no statistically significant difference between the two (p=0.75). However, Group B had significantly shorter mean tourniquet time (41.2 vs. 67.5 minutes), surgery time (58 vs. 79.1 minutes), and greater mean pronation movements (80.7° vs. 78.5°) compared to Group A (p<0.05).

In terms of complications, Group A had one case of delayed union of the ulna, managed with revision open reduction and Internal fixation with bone grafting, resulting in union and a 'GOOD' outcome as per the Grace and Eversmann scores at the final follow-up. Three cases of superficial wound infection were observed (two in open type I injuries and one in a closed fracture with abrasions), and all resolved with debridement and dressings. Five patients experienced stiffness in pronation and supination, but with physiotherapy, all achieved 'GOOD' to 'EXCELLENT' outcomes at the final follow-up. In Group B, three cases of delayed union of the ulna were noted; two cases healed with plaster immobilization and bone marrow aspirate injection, while the third case required open reduction and plate osteosynthesis with bone grafting. Ultimately all cases achieved union and 'EXCELLENT' outcome. One case of superficial infection at the proximal nail entry site was noted and it healed with local care. Four patients had stiffness in pronation-supination movements, all of whom improved with physiotherapy and achieved 'GOOD' outcomes based on the Grace and Eversmann scores at the final follow-up, with radiological union averaging 10-12 weeks in these cases.

Fernandez et al. [19] in their study indicated no significant difference in functional outcome between their groups, based on their scoring system comprising of X-rays, range of movements, and cosmesis for assessment. Based on this score, they reported very good to good outcomes in 91.5% of plating (n=19) patients and 97.7% of intramedullary nailing patients (n=45). Their plating group encountered two cases of refractures and a case with thumb hypoesthesia, while the intramedullary nailing group had a case of refracture, a case with pseudoarthrosis, three cases with thumb hypoesthesias, two cases with delayed fracture healings, and two patients with skin infections. Similarly, Ozkaya et al. [20], using Price criteria [21], reported 79% perfect, 14% good, and 7% fair results for their plating group (n=14), and 85% perfect and 15% good results for their intramedullary nailing group (n=21). Complications in their plating group included one case with delayed healing, one with non-union and another case with loss of thumb extension, and finally, two more cases of superficial wound infections, while their intramedullary nailing group experienced one case of delayed union, one patient with ulnar bursitis, two cases of ulnar neuropathy, one patient with irritation of the superficial radial nerve, and four patients with hardware migration postoperatively.

Shah et al. [22] observed no statistically significant differences in mean fracture union time or radial bow magnitude, with all patients achieving a functional range of movement in both groups. They concluded that intramedullary nailing (n=15) resulted in fewer complications, with their plating group (n=46) experiencing two cases of refractures and one case of neuropathy, and the intramedullary nailing group reporting a case of refracture, one patient with non-union, two cases of delayed unions, two patients with nail migrations, three cases of skin infections, and three patients with neuropathies. In contrast, Freese et al. [13] suggested that Open Reduction and Internal Fixation (ORIF) with plates and screws provided closer anatomical restoration and lower complication rates in adolescents compared to intramedullary nailing. Their plating group (n=32) had a 12% complication rate (wound dehiscence, superficial infection, finger flexion contracture, neurapraxia of superficial radial nerve), while the intramedullary nailing group (n=70) had a 29% complication rate (implant migration, malunion, pin site infections, neurapraxia, difficult implant removal, and non-union of ulna). Lastly, Abdulkareem et al. [23], also using Price criteria [21], reported 66.66% excellent and 33.33% good results for their plating group (n=21), 78.26% excellent and 21.74% good results for their intramedullary nailing group (n=23). Complications in their plating group included a case of compartment syndrome, one case of delayed union, four cases of edema, three patients with superficial infections, and one patient with deep infection, while their intramedullary nailing group reported one case of delayed union, two cases of superficial infections, and two cases with edema as a complication.

Fracture classification, tourniquet time, and time to union

Our study utilized the AO-OTA classification for classifying both-bone diaphyseal forearm fractures. We found 22A3 to be the most prevalent subtype (nine cases from Group A and 16 cases from Group B) in these types of fractures. The average time to union was 9.4 weeks for Group A and 8.5 weeks for Group B, respectively. The mean tourniquet times were found to be around 67.5 minutes and 41.2 minutes for Group A and B, respectively. Shah et al. [22], over 10 years, similarly found 22A3 to be the most common subtype (23 cases in their plating group and seven cases in their intramedullary nailing group) with time to union around 8.9 and 8.5 weeks for their plating group and intramedullary group, respectively, though tourniquet times were not included in their study. Freese et al. [13] also identified 22A3 as the common type (32 cases in their plating group and 68 cases in their intramedullary nailing group), with time to union around 8.2 and 9.7 weeks for their plating group and intramedullary group, respectively. They also reported tourniquet times of 86.6 and 45.7 minutes in the former and latter group, respectively. Comparatively, Reinhardt et al. [24] in their nine-year study also highlighted 22A3 as the most frequent subtype. They reported no significant difference in the union time between the two groups at three and six months postoperatively, with tourniquet times averaging 89.5 minutes for the plating group and 35.6 minutes for the intramedullary nailing, respectively. Table 6 summarizes the time to union and tourniquet time usage data reported in various studies.

**Table 6:** Comparison of the radiological union time and tourniquet time reported in various studies IM nailing: Intramedullary nailing; DCP: Dynamic Compression Plating

Study	Time to union in the DCP group	Time to union in the IM nailing group	Tourniquet time in the DCP group	Tourniquet time in the IM nailing group
Reinhardt et al. [24]	No difference in comparison with the IM nailing group (timing not reported)	No difference in comparison with the DCP group (timing not reported)	89.5 minutes	35.6 minutes
Freese et al. [13]	8.2 weeks	9.7 weeks	86.6 minutes	45.7 minutes
Present study	9.4 weeks	8.5 weeks	67.5 minutes	41.2 minutes

Age and sex distribution

In our study, participants in Group A and Group B had a mean age of 14.4 and 15.5 years, respectively, with a male-to-female ratio of 8:4 (19 male patients and nine female patients) and 8:3 (20 male patients and eight female patients) in both the groups. Table 7 shows the patient demographics and injury characteristics in forearm fractures across studies. 

**Table 7:** Comparison of age and sex distribution across studies IM nailing: Intramedullary nailing; DCP: Dynamic Compression Plating; M:F ratio: Male to female ratio. Data represented as the number of participants (n) and their mean age among both groups.

Study	DCP group (n; mean age)	IM nailing group (n; mean age)	DCP group (M: F ratio)	IM nailing group (M: F ratio)
Reinhardt et al. [24]	n=12 (14.5 years)	n=19 (12.5 years)	5:1	13:6
Shah et al. [22]	n=46 (14.1 years)	n=15 (13.3 years)	37:9	2:1
Smith et al. [25]	n=44 (12.7 years)	n=103 (10.6 years)	13:2	4:3
Freese et al. [13]	n=32 (14.2 years)	n=70 (12.1 years)	69:31	63:37
Present study	n=28 (14.4 years)	n=28 (15.5 years)	19:9	20:8

Mode of injury

In our study, RTA were the most frequent mode of injury (57.14% of cases), followed by falls (37.51%) and assault (5.35%) as the other modes of injury. Ozkaya et al. [20] (dynamic compression plating (n=14) and intramedullary nailing (n=21) cited falls (80%) as the most frequent mode of injury, followed by direct trauma (11.43%) and RTA (8.57%). Abdulkareem et al. [23] with dynamic compression plating (n=21) and intramedullary nailing (n=23) groups also observed injury secondary to falls (79.54%) as the leading cause followed by direct trauma (11.36%) and RTA (9.09%) in their study. Freese et al. [13] with 32 patients undergoing dynamic compression plating and 70 patients undergoing intramedullary nailing identified sports injury (48%) as the primary mode of injury, followed by trampoline injuries (11.8%), miscellaneous injuries (11.8%), and RTA (7.8%) as the other modes of injury. Kose et al. [26] with dynamic compression plating (n=11) and intramedullary nailing (n=21) groups also reported sports injuries (53.13%) as the most common cause, followed by injury due to falls (31.25%), and RTA (15.62%) in their study.

Study limitations

The study was conducted at a single center, limiting the generalizability of the findings. There was no long-term follow-up to assess the incidence of refractures or implant migration. Also, the evaluation regarding potential growth disturbances could not be included as well.

## Conclusions

Plate osteosynthesis provides rigid fixation, allows an early range of motion, and delivers both static and dynamic compression forces across the fracture site. Intramedullary nailing is also a safe, less invasive procedure when performed under closed reduction; however, the risk of compartment syndrome increases with multiple closed reduction attempts. While the operative duration for intramedullary nailing is shorter and offers better cosmesis, larger-sized nails can lead to risks such as nail incarceration, non-union, implant migration, and prominence.

Both methods of internal fixation are well-established and meet the primary goals of fracture reduction, union, and restoration of functional range of motion. Based on our study findings, intramedullary nailing can be a better option for simple fracture patterns, offering shorter surgery time and good cosmesis while preserving fracture biology. For comminuted, irreducible fractures, especially in older children approaching skeletal maturity, plate osteosynthesis is a better technique, as the expected degree of remodeling is reduced in such cases.
